# Early Sorafenib-Induced Toxicity Is Associated with Drug Exposure and UGTIA9 Genetic Polymorphism in Patients with Solid Tumors: A Preliminary Study

**DOI:** 10.1371/journal.pone.0042875

**Published:** 2012-08-13

**Authors:** Pascaline Boudou-Rouquette, Céline Narjoz, Jean Louis Golmard, Audrey Thomas-Schoemann, Olivier Mir, Fabrice Taieb, Jean-Philippe Durand, Romain Coriat, Alain Dauphin, Michel Vidal, Michel Tod, Marie-Anne Loriot, François Goldwasser, Benoit Blanchet

**Affiliations:** 1 Centre d'étude et de recours sur les inhibiteurs de l'angiogénèse, Paris, France; 2 Assistance Publique Hôpitaux de Paris, Hôpital Cochin, Unité de Cancérologie médicale, Paris, France; 3 Université Paris Descartes, Sorbonne Paris Cité, Paris, France; 4 Assistance Publique Hôpitaux de Paris, Hôpital Européen Georges Pompidou, Service de Biochimie, Unité Fonctionnelle de Pharmacogénétique et Oncologie Moléculaire, Paris, France; 5 Université Paris Descartes, INSERM UMR-S 775, Paris, France; 6 Assistance Publique Hôpitaux de Paris, Hôpital Pitié-Salpêtrière, Département de Biostatistiques, Paris, France; 7 Assistance Publique Hôpitaux de Paris, Hôpital Cochin, Unité Fonctionnelle de Pharmacocinétique et Pharmacochimie, Paris, France; 8 UMR8638 CNRS, UFR de Pharmacie, Université Paris Descartes, Sorbonne Paris Cité, Paris, France; 9 Pharmacie, Hôpital de la Croix-Rousse, Hospices civils de Lyon, Lyon, France; 10 EMR3738, Université de Lyon, Lyon, France; Istituto di Ricerche Farmacologiche Mario Negri, Italy

## Abstract

**Background:**

Identifying predictive biomarkers of drug response is of key importance to improve therapy management and drug selection in cancer therapy. To date, the influence of drug exposure and pharmacogenetic variants on sorafenib-induced toxicity remains poorly documented. The aim of this pharmacokinetic/pharmacodynamic (PK/PD) study was to investigate the relationship between early toxicity and drug exposure or pharmacogenetic variants in unselected adult outpatients treated with single-agent sorafenib for advanced solid tumors.

**Methods:**

Toxicity was recorded in 54 patients on days 15 and 30 after treatment initiation and sorafenib exposure was assessed in 51 patients. The influence of polymorphisms in *CYP3A5, UGT1A9, ABCB1* and *ABCG2* was examined in relation to sorafenib exposure and toxicity. Clinical characteristics, drug exposure and pharmacogenetic variants were tested univariately for association with toxicities. Candidate variables with p<0.1 were analyzed in a multivariate analysis.

**Results:**

Gender was the sole parameter independently associated with sorafenib exposure (p = 0.0008). Multivariate analysis showed that increased cumulated sorafenib (AUC_cum_) was independently associated with any grade ≥3 toxicity (p = 0.037); *UGT1A9* polymorphism (rs17868320) with grade ≥2 diarrhea (p = 0.015) and female gender with grade ≥2 hand-foot skin reaction (p = 0.018). Using ROC curve, the threshold AUC_cum_ value of 3,161 mg/L.h was associated with the highest risk to develop any grade ≥3 toxicity (p = 0.018).

**Conclusion:**

In this preliminary study, increased cumulated drug exposure and *UGT1A9* polymorphism (rs17868320) identified patients at high risk for early sorafenib-induced severe toxicity. Further PK/PD studies on larger population are warranted to confirm these preliminary results.

## Introduction

Sorafenib (Nexavar®) is a dual-action inhibitor that targets RAF/MEK/ERK pathway in tumor cells and tyrosine kinases VEGFR/PDGFR in tumor vasculature [1]. Sorafenib has demonstrated preclinical and clinical activity against several tumor types [1], [2]. It is currently approved for the treatment of renal cell and unresectable hepatocellular carcinomas. Hand-foot skin reaction (HFSR), diarrhea, asthenia and hypertension are the most frequent adverse reactions of clinical importance in sorafenib-treated patients [3]. These toxicities are often manageable with ancillary treatments, however they may lead to drug discontinuation or dose reduction that can decrease the potential life-prolonging benefits of sorafenib [4]. Due to the widespread use of sorafenib and the associated risk of side effects, the question of defining subgroups of patients susceptible to sorafenib-related toxicities is of crucial clinical importance.

Sorafenib is metabolized primarily in the liver and undergoes oxidative metabolism mediated by cytochrome P450 3A4 isoform (CYP3A4), as well as glucuronidation mediated by uridine diphosphate glucuronyl transferase 1A9 (UGT1A9) [5]. Given that CYP3A4 and CYP3A5 exhibit significant overlap in substrate specificity, the CYP3A5 pathway may also be involved in the metabolism of sorafenib. The large variations in the pharmacokinetics of sorafenib [5] may be due to patients' characteristics and/or genetic backgrounds. Food and albuminemia have been identified as factors that may contribute to these variations [6], [7], even if conflicting data have been published [8], [9]. So far, as regards pharmacogenetic variants, a single investigation has evaluated the effect of genotype with respect to CYP3A4*1B, CYP3A5*3C, UGT1A9*3 and UGT1A9*5 on sorafenib pharmacokinetics [8]. None of these pharmacogenetic variants was associated with sorafenib pharmacokinetics. However, the impact of two specific single-nucleotide polymorphisms (SNPs) in the gene promoter region of *UGT1A9* (−275 T>A, −2152 C>T) was not evaluated in that study [8] although the −2152 C>T and −275 T>A SNPs, occurring at the frequency of 10–17% in the Caucasian population [10], are correlated with higher hepatic expression of UGT1A9 and increased in vitro glucuronidation activity [11]. These two SNPs have already been associated with significantly lower exposure to mycophenolic acid in renal recipients [10], and thereby may also contribute to the variability in sorafenib exposure. Finally, ATP-binding cassette (ABC) transporters such as P-glycoprotein (P-gp encoded by *ABCB1*) and breast cancer resistance protein (BCRP encoded by *ABCG2*) are known to play an important role in the pharmacokinetics of different TKIs [5]. Thus, sorafenib is efficiently transported by BCRP and moderately by P-gp [12], [13]. Yet, the influence of *ABCG2* and *ABCB1* on sorafenib exposure remains to be established in cancer patients.

In contrast with other TKIs used in the treatment of solid tumors [14]–[23], little is known about the influence of drug exposure and pharmacogenetic variants on interindividual variability in early sorafenib-induced toxicity. The main goal of this exploratory pharmacokinetic/pharmacodynamic (PK/PD) study was to determine whether sorafenib exposure and pharmacogenetic variants (nine common SNPs of *CYP3A5, UGT1A9, ABCB1* and *ABCG2)* may predispose to interindividual variability in common early acute sorafenib-induced toxicities in adult outpatients treated with single-agent sorafenib for advanced solid tumors.

## Materials and Methods

### Ethics statement

The investigational review board “Comité de Protection des Personnes d'Ile de France” approved the study protocol (N°AT140); all patients provided written informed consent and approved the sampling and pharmacogenetic analysis in compliance with the ethical principles of the revised Declaration of Helsinki (2008) and with French regulations.

### Patients' selection

From May 2008 to May 2011, a total of 92 consecutive patients with advanced or metastatic solid tumors were treated with single agent sorafenib at the Cochin Teaching Hospital in Paris, France. The study population represents a subgroup of these patients for whom genotyping was accepted and performed. All patients met the following criteria: age ≥18 years; presence of clinically and/or radiologically assessable disease. Patients with brain metastases were included.

### Treatment plan and toxicity assessment

Patients were started on sorafenib on a twice-daily (bid) schedule (200 mg bid or 400 mg bid depending on their status). Adverse events were reported and graded according to the National Cancer Institute Common Terminology Criteria (NCI-CTCAE) on days 15 and 30 after treatment initiation. Diarrhea and HFSR were graded according to the NCI-CTCAE 3.0 version [24], while hypertension was retrospectively graded according to the 4.0 version [25]. In case of grade 4 hematological toxicity or grade 3 or 4 non-hematological toxicity, treatment was discontinued until side effects recovered to grade 1, or returned to baseline. Subsequent dose reductions were left at the discretion of the treating physician. At each follow-up visit, blood samples were drawn into 5-mL lithium heparinized Vacutainer tubes to determine plasma concentrations of sorafenib. After centrifugation at 3,000 rpm for 5 minutes at 4°C, plasma was transferred to propylene tubes and stored at −20°C until assay of plasma sorafenib concentrations.

### Sorafenib pharmacokinetic analysis

Sorafenib concentrations in plasma were determined using a method previously published by our research group [26]. The calibration was linear in the range of 0.5–20 mg/L. The accuracy, within-assay and between-assay precision of this method were 96.9–104.0%, 3.4–6.2% and 7.6–9.9%, respectively. A specific Bayesian estimator developed in our institution [27] allowed estimating individual sorafenib area under the plasma concentration-time curve from 0 to 12 hours (AUC) and the average plasma sorafenib concentration (C_av_) over 12 hours. This Bayesian estimator is based on a 1-compartment model with saturated absorption, first-order intestinal loss and linear elimination.

### DNA extraction and Genotyping analysis

All enrolled patients were genotyped for 9 polymorphisms in 4 genes potentially involved in the pharmacokinetics of sorafenib. Genotyping was performed in the central laboratory (Hôpital Européen Georges Pompidou, Paris). Genomic DNA was extracted from peripheral blood leukocytes using the QiaAmp DNA mini Kit (Qiagen, Courtaboeuf, France) according to the recommendations of the manufacturer. Following SNPs were identified using Taq Man® Drug Metabolism Genotyping Assays (Applied Biosystems, Courtaboeuf, France): 6986 A>G (rs 776746, C__26201809_30) for *CYP3A5*, 3435 G>T (rs 1045642, C___7586657_20) and 2677 G>T/A (rs 2032582, C_11711720D_40 and C_11711720C_30) for *ABCB1*, −275 T>A (rs 6714486, C__27843087_10), −2152 C>T (rs17868320, C__34418857_10), 98 T>C (rs 72551330, C__64627083_10) for *UGT1A9*, 421C>A (rs 2231142, C__15854163_70) and 1143 C>T (rs 2622604, C__9510352_10) for *ABCG2*. Genotype for *ABCG2* 34G>A was determined by sequencing. Its cycling conditions and primers are available on request. Its sequencing was performed on an ABI Prism Genetic Analyzer System 9700 (Applied Biosystems).

Due to DNA quantity or poor DNA quality genotyping, success rates were 98% for CYP3A5*3, *UGT1A9* and *ABCB1* SNPs and 94% for *ABCG2* SNPs

### Statistical analysis

For descriptive statistics, qualitative variables were expressed in numbers and percentages and quantitative ones in medians [inter-quartile intervals]. Glomerular filtration rate (GFR) and lean body mass (LBM) were estimated using the Modification of the Diet in Renal Disease (MDRD) formula and the Green's formula, respectively [28], [29]. To identify the covariates influencing the pharmacokinetics of sorafenib on day 15 after treatment initiation (i.e. once the pharmacokinetic steady-state was reached), the individual absolute AUCs estimated were normalized to a dose of 400 mg sorafenib twice daily. Then, the relationships between the dose-standardized AUC and other variables were tested using two-sample Wilcoxon tests or Kruskal-Wallis tests for qualitative variables with two modalities or more than two modalities, respectively, and by Spearman rank correlation coefficient tests for quantitative ones. The following variables were tested: age, gender, ECOG status, weight, body mass index (BMI), LBM, primary tumor site, polymorphisms of *CYP3A5* (rs776746), *UGT1A9* (rs178868320, rs6714486, rs72551330), *ABCB1* (rs2231137, rs1045642), *ABCG2* (rs2231137, rs2231142, rs2622604), alanine aminotransferase (ALT), aspartate aminotransferase (AST), alkaline phosphatase (ALP), gamma-glutamyl transpeptidase (GGT), bilirubin, albumin, C-Reactive Protein, creatinine and MDRD GFR. A stepwise multiple linear regression was then performed with variables significant with p<0.10 in the first step included in the regression and variables significant with p<0.05 kept in the final model. For the analysis of sorafenib-induced toxicities in the first month of treatment, we used dichotomous end points expressed as any toxicity ≥ grade 3 (yes or no), or grade ≥2 HFSR (yes or no), or grade ≥2 diarrhea (yes or no), or grade ≥2 hypertension (yes or no). The cumulated AUC between day 0 and day 30 was calculated as the sum of two areas, (0–15 days) and (15–30 days) which were estimated using the trapezoidal rule. The area was multiplied by 24 to express the result in mg/L.h. The cumulated AUC (AUC*_cum_*) on day 30 was calculated according to the following formula: 
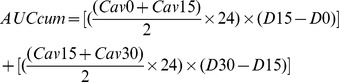
where Cav and D are average plasma sorafenib concentrations over 12 hours and days, respectively. On day 0, C*_av0_* was fixed at 0 mg/L.

The univariate analysis of toxicity risk factors was carried out using two sample Wilcoxon tests for quantitative variables and Chi square or Fisher exact tests for qualitative ones. Fisher exact tests were performed as soon as at least one expected value was lower than 5. The following variables were tested: age, gender, ECOG status, weight, BMI, LBM, primary tumor site, polymorphisms of *CYP3A5* (rs776746), *UGT1A9* (rs178868320, rs6714486, rs72551330), *ABCB1* (rs2231137, rs1045642), *ABCG2* (rs2231137, rs2231142, rs2622604), ALT, AST, ALP, GGT, bilirubin, albumin, CRP, creatinine, MDRD GFR, daily sorafenib dose, daily dose of sorafenib (in mg or expressed as mg/m^2^), absolute sorafenib AUC and AUC*_cum_*. The multivariate analysis was performed by a stepwise logistic regression. Variables with p-values<0.10 in the univariate analysis were included in the regression and variables with a Wald test p-value<0.05 were kept in the final model. All the tests were two-sided, and they were considered significant when p-values were <0.05. The receiver operating characteristic (ROC) curve was constructed to examine the value of an AUC*_cum_* threshold predicting a toxicity of grade ≥3 in the first month of treatment. The diagnostic accuracy values of sensitivity and specificity were determined, as well as the positive predictive value (PPV) and the negative predictive value (NPV). Computations were performed using the SAS V9 statistical package (SAS institute, Cary, NC).

## Results

### Patients

Fifty-eight adult patients with advanced solid tumors were enrolled in this monocentric study. Four patients had to be excluded from the analysis: two never began sorafenib treatment (one for severe sepsis, the other because he died before treatment initiation), one patient did not come to the first follow-up visit and thereafter, and finally one patient for lack of acceptable genotyping success rate due to poor DNA quality. Overall, 54 patients were assessable for toxicity. The clinical and biological baseline characteristics of these patients are summarized in Table 1. During the whole follow-up period, the median sorafenib dose was 800 mg/day (range 200–1200 mg/day). Regarding sorafenib exposure, the median absolute sorafenib AUC was 62.0 [45.4–91.8] mg/L.h and 61.0 [31.6–75.4] mg/L.h on days 15 and 30, respectively. The median absolute AUC in females was significantly greater than that observed in males on day 15 (78.3 [60.4–124.4] vs 52.4 [42.6–81.4] mg/L.h, respectively; p = 0.006) and day 30 (82.2 [57.7–108.9] vs 55.2 [31.4–72.5] mg/L.h respectively; p = 0.0078).

**Table 1:** Clinical and biological characteristics of study group.

Characteristics	N = 54
**Demographic data**	
Gender, n (%)	
Male	38 (70)
Female	16 (30)
Age in years	64 [58–76]
BSA (m^2^)	1.9 [1.7–2.0]
BMI (kg/m^2^)	24.0 [22.4–29.0]
LBM^a^ (kg)	53.1 [50.2–58.6]
ECOG performance status, n (%)	
0	18 (33)
1	22 (41)
≥2	14 (26)
**Primary sites, n (%)**	
Hepatocellular carcinoma	20 (37)
Melanoma	13 (24)
Differenciated thyroid cancer	12 (22)
Renal cell carcinoma	6 (11)
Other	3 (6)
**Baseline biological data**	
AST (UI/L)	36 [27–68]
ALT (UI/L)	34 [21–51]
ALP (UI/L)	87 [66–186]
GGT (UI/L)	57 [25–182]
Bilirubin (µmol/L)	10 [7]–[13]
Albumin (g/L)	39 [34–41]
CRP (mg/L)	7 [3]–[20]
Creatinine Clearance^b^ (mL/min)	69.6 [54.6–82.2]

ALP, alkaline phosphatase; ALT, Alanine aminotransferase; AST, Aspartate aminotransferase; BMI, body mass index; BSA, body surface area; CRP, C-reactive protein; ECOG, Eastern Cooperative Oncology Group; GGT, Gamma-glutamyl transpeptidase; LBM, Lean Body Mass.

Results are expressed as median [interquartile range].

a Lean body mass was estimated by using the Green's formula [29].

b Creatinine clearance was estimated by using Modification of Diet in Renal Disease (MDRD) formula [28].

### Genotype distribution

Table 2 presents the genotype distribution of the different polymorphisms. Variant allele frequencies estimated on the entire series of 54 patients were in accordance with those observed in the Caucasian population. Genotypes were distributed according to the Hardy Weinberg equilibrium except for *CYP3A5* and *UGT1A9* (98 T>C). For *CYP3A5*, a greater than expected number of the variant CYP3A5*1*1 was observed. However this deviation was not observed when considering only our Caucasian patients, who represented 87% of our population. None of the patients carried the variants for *UGT1A9* 98 T>C.

**Table 2:** Genotype distribution and minor allele frequency among the 54 included patients.

Gene	Identification	SNPs	Genotype			
	number		wt/wt	wt/m	m/m	Minor allele
			n (%)	n (%)	n (%)	frequency
**CYP3A5**						
	rs776746	6986 A>G	4 (7)	11 (20)	39 (73)	0.185^a^
**UGT1A9**						
	rs17868320	-2152 C>CT	50 (92)	4 (8)	0 (0)	0.075
	rs6714486	-275 T>A	48 (90)	5 (10)	0 (0)	0.096
	rs72551330	98 T>C	54 (100)	0 (0)	0 (0)	0
**ABCB1**						
	rs2032582	3435 C >T	17 (32)	25 (46)	12 (22)	0.452
	rs1045642	2677 G >T/A	22 (41)	22 (41)	10 (18)	0.415
**ABCG2**						
	rs2231137	34 G >A	41 (79)	10 (19)	1 (2)	0.117
	rs2231142	421 C>A	42 (81)	8 (15)	2 (4)	0.117
	rs2622604	1143 C>T	32 (61)	16 (31)	4 (8)	0.235

m, mutant allele, SNP, Single-Nucleotide Polymorphism; wt, wild-type allele.

a the less common allele for CYP3A5 was the A-allele (wild type allele) in our predominantly Caucasian population

### Factors influencing sorafenib exposure

**Figure 1:**
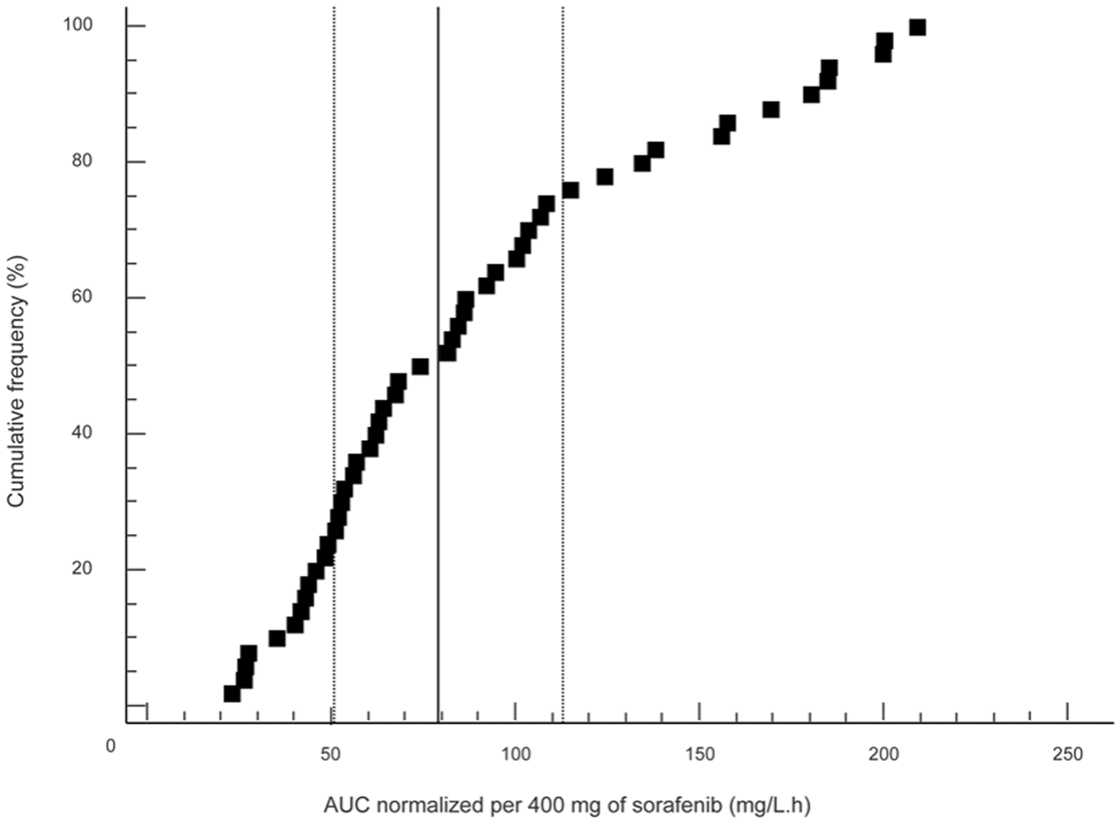
Distribution of sorafenib exposure (AUC) on day 15 after treatment initiation (n = 51). AUC was normalized per 400 mg of sorafenib. The solid line represents the median. The dotted lines represent 25^th^ and 75^th^ percentiles.

There was wide interindividual variability in dose-normalized exposure to sorafenib on day 15 (*n* = 51; CV = 58.0%), and the median dose-normalized AUC was 78.0 [51.0–114.8] mg/L.h (Figure 1). The median dose-normalized AUC in females was 2.1-fold greater than that observed in males (136.0 vs 64.8 mg/L.h, respectively, p = 0.0008) (Table 3). CYP3A5*1*1 carriers presented a greater exposure than CYP3A5*3*3 carriers (136.0 mg vs 67.4 mg/L.h, respectively), related to the predominance of females in CYP3A5*1*1 carriers (80%) compared to CYP3A5*3*3 (29%) carriers. Patients with the *ABCG2* 1143 TT genotype exhibited a greater exposure compared to those with the *ABCG2* 1143 CC genotype (131.8 vs 82.4 mg/L.h, respectively). The multiple linear regression showed that gender (p = 0.0008) was the single parameter independently associated with the dose-normalized exposure to sorafenib on day 15 after treatment initiation.

**Table 3:** Genetic and non-genetic factors influencing exposure to sorafenib on day 15 after treatment initiation.

	Univariate analysis			Linear regression
Variable	Sorafenib AUC a	Spearman's	P value	P value
	(mg/L.h)	coefficient (rho)		
Gender			**0.011**	**0.0008**
Female	136.0 [60.4–182.5]			
Male	64.8 [45.4–94.2]			
*CYP3A5* 6986 A>G			0.063	NS
GG (*3/*3)	67.4 [51.0–124.0]			
AG (*1/*3)	75.0 [43.1–90.4]			
AA (*1/*1)	136.0 [117.8–168.8]			
*ABCG2* 1143 C>T			0.093	NS
CC	82.4 [52.2–139.9]			
CT	64.8 [41.3–103.1]			
TT	131.8 [96.0–170.1]			
LBM^b^ (kg)		−0.28	0.053	NS
Creatinine (µmol/L)		−0.26	0.076	NS

AUC, area under the curve; LBM, Lean Body Mass

Results are expressed median [interquartile range]

a AUC was dose-normalized per 400 mg of sorafenib

b Lean body mass was estimated by using the Green's formula [29]

Data were available for 51 patients

### Toxicity

The most common grade 2–3 events were HFSR in 22 (41%) of the 54 patients, hypertension in 20 (38%) and diarrhea in 5 (9%) (Table 4). Twenty-two patients (41%) experienced grade 3 or 4 toxicity on days 15 or 30: HFSR (n = 15, 68%), followed by hypertension (n = 6, 27%), and diarrhea (n = 1, 5%). One patient exhibited grade 3 hypertension on day 15, followed by a grade 3 HFSR on day 30. Nineteen patients (35%) required dose reductions (n = 9) or interruption of treatment (n = 10) because of toxic effects over the study period. Finally, there was no toxic death in relation with sorafenib.

**Table 4:** Distribution of increased toxicity grades occurring during the first month of therapy.

Toxicity by grade	Occurrence during first month of treatment n (%)
**Hand-foot skin reaction**	
No	17 (31)
Yes	37 (69)
<2	15
≥2	22
**Hypertension**	
No	28 (52)
Yes	25 (46)
<2	5
≥2	20
NA	1
**Diarrhea**	
No	37 (69)
Yes	17 (31)
<2	12
≥2	5

NA: not assessed

### Risk factors for any grade ≥3 toxicity and grade ≥2 HFSR, diarrhea and hypertension

Table 5 shows that increased AUC*_cum_* was the sole parameter independently associated with any grade ≥3 toxicity (OR: 1.07; 95% CI, 1.01–1.12; p = 0.037). Regarding specific toxicities, female sex was identified as an independent risk factor for developing grade ≥2 HFSR (OR: 5.26; 95% CI, 1.33–20.0; p = 0.018), and the presence of the mutant T allele in *UGT1A9* −2152 C>T for grade ≥2 diarrhea (OR: 14.33; 95% CI, 1.46–140.50; p = 0.015) (Table 5). In contrast, no independent risk factor was identified for the development of grade ≥2 hypertension.

**Table 5:** Risk factors associated with sorafenib-induced toxicity, defined as any toxicity ≥grade 3 or hand foot skin reaction, hypertension and diarrhea grade ≥2.

	Univariate analysis			Multivariate analysis	
Variable	No	Yes	*p-*value	OR (95% CI)	*p-*value
***Any toxicity ≥ grade 3***					
Gender			0.024		NS
Female, n (%)	5 (33.3)	10 (66.7)			
Male, n (%)	25 (67.6)	12 (32.4)			
*CYP3A5* 6986 A>G, n(%)			0.05		NS
GG (*3/*3)	24 (63)	14 (37)			
AG (*1/*3)	6 (67)	3 (33)			
AA (*1/*1)	0 (0)	4 (100)			
Cumulated sorafenib AUC (mg/L.h)	2,250	3,499	0.034	**1.07 (1.01**–**1.12)**	**0.019**
	[1,845–2,858]	[2,070–4,019]			
***Hand Foot Skin Reaction ≥*** ** ***grade 2***					
Gender			0.024	**5.26 (1.33**–**20.0)**	**0.018**
Female, n (%)	5 (33.3)	10 (66.7)			
Male, n (%)	25 (52.0)	12 (48.0)			
ECOG PS	1 [1]–[2]	1 [0–1]	0.043		NS
Cumulated sorafenib AUC (mg/L.h)	2,250	3,308	0.042		NS
	[1,789–2,858]	[2,070–4,365]			
***Diarrhea ≥ grade 2***					
*UGT1A9* −275 T>A			0.043		NT*
wt/wt, n (%)	43 (93)	3 (7)			
wt/m, n (%)	2 (50)	2 (50)			
m/m, n (%)	0 (0)	0 (0)			
*UGT1A9* −2152 C>T			0.045	**14.33 (1.46**–**140.50)**	**0.015**
wt/wt, n (%)	42 (93)	3 (7)			
wt/m, n (%)	2 (50)	2 (50)			
m/m, n (%)	0 (0)	0 (0)			
***Hypertension ≥ grade 2***					
Albumin (g/L)	37 [34]–[40]	41 [37–42]	0.06		NS
Daily dose of sorafenib (mg/m^2^)	392 [232–434]	220 [177–394]	0.053		NS

AUC, area under the curve; BMI, body mass index; ECOG PS, Eastern Cooperative Oncology Group Performance Status; m, mutant allele; NS, not significant; NT, not tested; OR, Odds ratio; Wt, wild-type allele.

Quantitative results are expressed as median [interquartile range]

* Heterozygotous patients (wt/m) for *UGT1A9*–2152 C>T and *UGT1A9*–275 T>A were the same; therefore the polymorphism for *UGT1A9*–275 T>A was not tested in the multivariate analysis

### Determination of a threshold for sorafenib AUC_cum_

**Figure 2:**
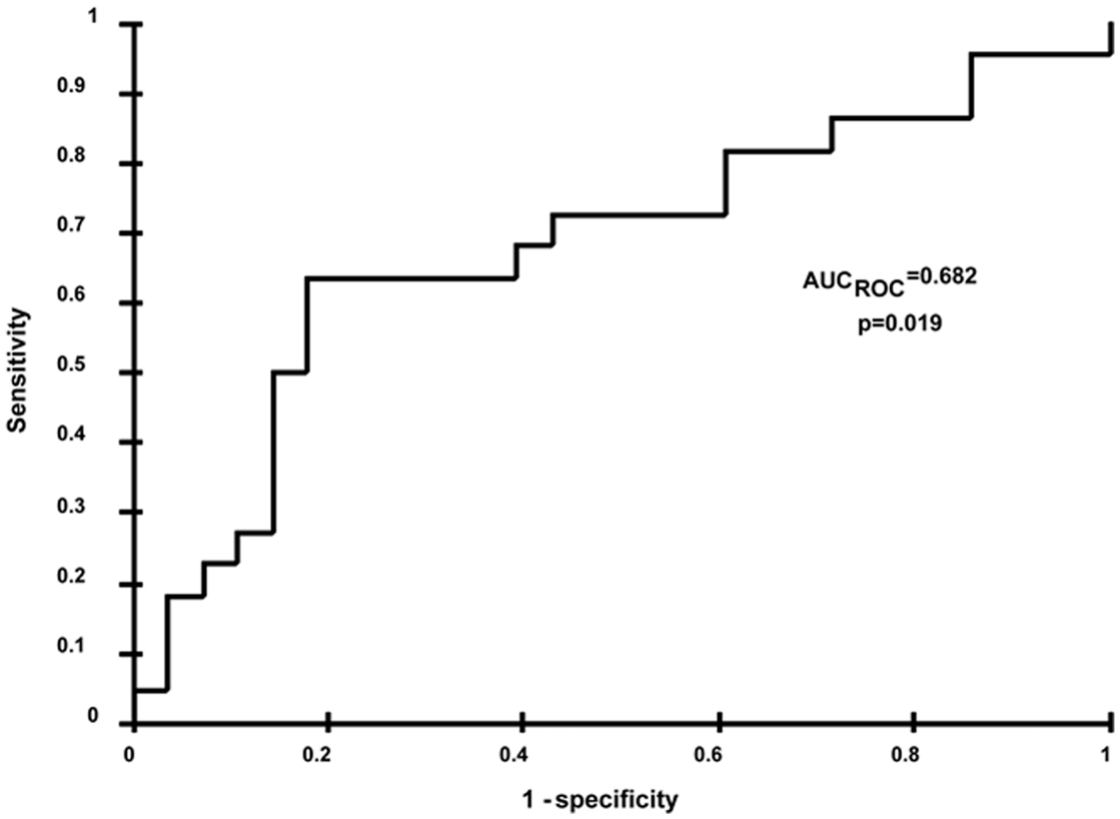
Receiver operating characteristic (ROC) curve estimates for the 51 patients. Sensitivity and 1 minus specificity for the risk of any grade ≥3 toxicity in the first month of treatment are shown. The proposed threshold cutoff value is 3,161 mg/L.h. AUC_ROC_ is the area under the ROC curve. The reported p value was calculated to test the null hypothesis that the AUC = 0.50.

Using the ROC curve (Figure 2), the AUC*_cum_* threshold predicting a toxicity of grade ≥3 was 3,161 mg/L.h with the area under the curve at 69% (CI 95%, 61–78%; p = 0.018) in our population. Considering the threshold value of 3,161 mg/L.h, PPV and NPV were 75.8% and 63.3%, respectively.

## Discussion

Identifying predictive biomarkers of drug response is of key importance to improve therapy management and drug selection in cancer therapy. Data regarding determinants of sorafenib-induced toxicity still remain scarce: age for therapy discontinuation in Japanese patients [30], cumulative sorafenib dose, ECOG performance status and female gender for HFSR [31], [32] . Besides, a thorough understanding of the dose-concentration-effect relationship of drug-induced toxicity is essential for the clinical use of sorafenib. Herein, we have shown for the first time that cumulated sorafenib exposure is determining for the development of any grade ≥3 toxicity and *UGT1A9*- 2152 T allele for grade ≥2 diarrhea.

The present results confirm the large interindividual variability in sorafenib exposure [8], [27], [33]. In disagreement with two population pharmacokinetic studies [8], [27], we observed an influence of gender on sorafenib dose-normalized exposure on day 15 after treatment initiation since females were overexposed compared to males. The greater exposition to sorafenib in females may be related in part to their lower median LBM compared to that of males (45.6 vs 55.6 kg, respectively; p = 0.0001). Indeed, several investigations have documented a positive relationship between LBM and total clearance of anticancer drugs such as epirubicin and carboplatin [34], [35]. In this context, the low LBM in females may result in a decreased total clearance of sorafenib and therefore in an increased drug exposure compared to males. Additionally, the inverse relationship between LBM and sorafenib exposure displayed by the univariate analysis supports this hypothesis. Further investigations are required to clarify the influence of LBM on sorafenib pharmacokinetics by using a computed tomography-scan, which is the benchmark method to determine LBM in cancer patients [36].

Regarding the pharmacogenetic approach, no genetic variants of metabolizing enzymes and efflux transporters were related to sorafenib exposure in the present study. The limited sample size of our cohort probably rendered the detection of any significant effect of pharmacogenetic variants (especially for *UGT1A9* and *ABCG2*) on sorafenib exposure difficult. Regarding *ABCG1* and *ABCG2*, our results are in line with two pharmacokinetic investigations carried out in mice [13], [37]. Indeed, the latter studies suggest that P-gp and BCRP would be expected to have only a minor effect on sorafenib pharmacokinetics in cancer patients. Besides, a recent population pharmacokinetic study in 111 patients has reported the lack of any effect of genotype with respect to CYP3A4*1B, CYP3A5*3C, UGT1A9*3 and UGT1A9*5 on the disposition of sorafenib [8]. Taken together, these results suggest that investigating the influence of pharmacogenetic variants on sorafenib pharmacokinetics in cancer patients may be particularly complex due to the two metabolic pathways (UGT1A9 and CYP3A4), the minor frequency of polymorphisms in *UGT1A9* that therefore requires a larger cohort of patients, and finally the confounding effect of the poor solubility of sorafenib in the gastrointestinal medium that may contribute to a large intra- and interindividual variability in bioavailability.

In the present cohort of patients, toxicities occurring during the first month of sorafenib therapy were used as outcome measures for different reasons. Firstly, the screening of pharmacogenetic variants within the routine pre-therapeutic evaluation is likely to be more relevant to predict early severe toxicities. Secondly, patients from a “real life” population are frailer compared to those selected in clinical trials, and therefore more vulnerable to developing severe toxicities. Finally, signs of clinical deterioration due to the disease progression over time could be misinterpreted and could interfere with the drug-induced toxicity outcome.

Herein, we provide the first evidence that an increased cumulated sorafenib exposure is associated with an increased risk of grade ≥3 toxicity in the first month of treatment. This result is in line with other PK/PD studies that have documented, in patients treated for solid tumors, an association between TKI exposure and drug-induced toxicity such as skin rash for erlotinib [20]–[22], hematologic toxicity and asthenia for sunitinib [23], and hematologic toxicity for imatinib [38]. Besides, a cumulated sorafenib AUC of 3,161 mg/L.h seems to be a significant threshold for developing any grade ≥3 toxicity. However, further studies with larger cohorts are required to determine optimal threshold value before its use in daily clinical routine to prevent the occurrence of grade ≥3 toxicities and thereby to avoid a transient interruption or a definitive discontinuation of sorafenib therapy.

In contrast with other TKIs [14], [17]–[19], no pharmacogenetic determinant has yet been identified for the development of sorafenib-induced toxicity. The present results highlight that the risk of sorafenib-induced grade ≥2 diarrhea strongly increases when the T allele in *UGT1A9* −2152 C>T is present (OR 14.33). This result is particularly interesting because severe diarrhea could result in a significant decrease in the bioavailability of sorafenib, which might lead to a decreased systemic drug exposure and possibly to a lesser anti-tumor efficacy. From a pathophysiological point of view, sorafenib-induced diarrhea may be related to the glucuronidation of the carboxylic acid M6, which is the major sorafenib metabolite found in feces (19.1% of the dose) [6]. Thus, the increase in intestinal expression of UGT1A9, and hence in glucuronidation activity related to the *UGT1A9* −2152 C>T polymorphism, may cause a significant biostranformation of the carboxylic acid M6 to reactive acyl glucuronide metabolites, which are known to damage enterocytes and cause diarrhea [39]. This hypothesis could explain the fact that this polymorphism is associated with severe diarrhea without any impact on sorafenib systemic exposure. However, due to the small sample size and the relatively low frequency of *UGT1A9* variants, further studies are required to confirm the association between the *UGT1A9* 2152 C>T polymorphism and diarrhea.

Hand-foot skin reaction was the most prevalent toxicity in the present study, which is in consistence with the shorter delay of occurrence of this toxicity compared to diarrhea and hypertension. In the present cohort, the risk for developing grade ≥2 HFSR in the first month of sorafenib therapy was approximately 5-fold greater in females compared to males (OR 5.26). This result is in agreement with a recent study which documented female gender as an independent risk factor for developing grade ≥2 HFSR [32]. Finally, the present study highlights for the first time that greater sorafenib exposure in females compared to males over the first month of therapy may be a determining factor of sorafenib-induced HFSR in females.

In a context of severe toxicity, decisions regarding sorafenib therapy (need for treatment interruption, dose reduction or change in therapy) are currently based on the overall clinical situation. In the present study, cumulated sorafenib exposure and not the daily dose of sorafenib was significantly associated with the occurrence of grade ≥3 toxicity, which suggests that drug plasma monitoring could help clinical decision-making in patients suffering from severe sorafenib-related adverse events. Besides, drug plasma monitoring may be helpful in other clinical scenarios, especially when a poor adherence is suspected in non-responders, or in the context of a pharmacokinetic drug-drug interaction [40]. Finally, an appropriately designed study is currently under way in our institution to explore the relationship between drug exposure and anti-tumor efficacy.

In conclusion, our results give the first evidence of an association between cumulated sorafenib exposure and the development of any grade ≥3 toxicity during the first month of treatment. Our preliminary findings also suggest that *UGT1A9* genotyping before treatment initiation may help to identify patients at higher risk for grade ≥2 diarrhea, and could support an *a priori* dose reduction in order to avoid such toxicity. However, much larger replication studies are needed to confirm the influence of UGT1A9 genotype on the individual susceptibility towards early sorafenib-induced toxicity. Taken together, these data suggest the clinical utility for individualized dosing regimens based on sorafenib exposure and genotype to improve drug tolerance and optimize drug selection.
